# Editorial: Patient education in the treatment of chronic pain

**DOI:** 10.3389/fpain.2025.1759323

**Published:** 2026-01-13

**Authors:** Shirin Modarresi, Erfan Shafiee

**Affiliations:** 1 School of Rehabilitation Science, McMaster Univeristy, Hamilton, ON, Canada; 2 Department of Health and Rehabilitation Science, Western University, London, ON, Canada; 3 Ruyere Research Institute, Ottawa, ON, Canada

**Keywords:** chronic pain, pain neuroscience education (PNE), patient education, self-management, multimodal pain management, health literacy, rehabilitation engagement, patient-centered care

Chronic pain continues to challenge patients and clinicians alike globally. One thing that has become clear is that education is no longer a minor add-on to conventional care. Rather, it is becoming a central part of how people make sense of their pain, engage with rehabilitation, and regain function. In this Research Topic, we aimed to explore the evolving landscape of patient education in chronic pain management. Across the five contributions included in this Topic, one message is clear: patient education is not a static or singular intervention but rather a dynamic, multifaceted tool that shapes how people understand, interpret, and respond to their pain experiences.

A prominent theme was the integration of technology to enhance engagement and accessibility. The study by Mardon et al. entitled “*The acceptability, feasibility, and usability of a virtual reality pain education and rehabilitation program for Veterans: a mixed methods study*” demonstrates that a Virtual Reality–based Pain Science Education (VR-PSE) program is both feasible and acceptable for veterans with persistent pain. What stood out was not only that participants found it feasible and easy to use, but also how much they valued the immersive, adaptable format. At the same time, the authors reminded us of something important: technology only works when it fits seamlessly into clinical care, guided by good screening and strong clinician–patient rapport.

The need for tailored educational content was emphasized in the second study by Mardon et al. titled “*Pain science education concepts for pelvic pain: an e-Delphi of expert clinicians”*. Here, an international panel of multidisciplinary experts identified 125 key learning concepts specifically for females with persistent pelvic pain, providing a framework for the development of condition-specific curricula. This study illustrates that patient education must be both evidence-based and relevant to the lived experiences of different patient populations.

Couëpel et al. in their study titled “*Effect of physical activity education on shoulder girdle pain and muscle strength in participants with fibromyalgia*” provide evidence that even brief educational interventions can have a tangible impact on pain perception. A five-minute video delivering patient education significantly reduced middle deltoid pain following a shoulder fatigue task among participants with fibromyalgia. These findings highlight the potential of patient education to modulate pain experience and enhance self-efficacy, even over short exposure periods.

Cuenca-Martinez et al. in their study titled “*Pain neuroscience education in patients with chronic musculoskeletal pain: an umbrella review*” underscore the importance of integrating education with other therapeutic interventions demonstrating that Pain Neuroscience Education (PNE) is most effective when delivered alongside exercise or multimodal approaches. Standalone PNE showed limited or inconsistent effects on pain, disability, or psychosocial outcomes, emphasizing that education alone is rarely sufficient to alter the complex interplay of factors driving chronic pain. This insight aligns with broader clinical observations: patient education is not simply information provision but a tool that enhances the efficacy of active rehabilitation.

Finally, the study by Moseley titled “*From didactic explanations to co-design, sequential art and embodied learning: challenges, criticisms, and future directions of patient pain education*” provides a critical and forward-looking perspective on the evolution of Pain Science Education. Over the past two decades, educational strategies have moved from didactic approaches to interactive, patient-centered models that incorporate co-design, multimedia learning, and embodied pedagogical techniques. This evolution reflects a growing recognition that improving patients’ conceptual understanding of pain is a key determinant of functional improvement and quality of life. By engaging patients actively in their learning, clinicians can foster deeper understanding, promote behavioral change, and support more effective self-management.

Taken together, these five papers paint a picture of a field that is dynamic, innovative, and increasingly patient-centered. They remind us that good pain education is interactive, context-specific, and best delivered alongside other evidence-based treatments. They also highlight the promise of emerging tools like VR and multimedia content, which can make education more engaging and accessible, especially for groups with unique needs.

Looking forward, there is a need for larger-scale trials, long-term evaluations, and importantly the co-creation of educational content with patients. As the field advances, there is an opportunity to refine how education is delivered, expand its reach through technology, and embed it meaningfully within broader pain management strategies. By doing so, we can continue to empower patients with chronic pain, enhance their self-efficacy, and ultimately improve clinical outcomes and quality of life.

In conclusion, this Research Topic provides a compelling snapshot of the current state and future directions of patient education in chronic pain. The diverse contributions emphasize that patient education has moved far beyond handing out information sheets. It's becoming an active, creative, and essential part of chronic pain care, one that has real potential to shape patient confidence and experience, self-management, and long-term outcomes.

